# Profile of people treated for hepatitis C: a descriptive study, Pelotas, Brazil, 2023

**DOI:** 10.1590/S2237-96222026v35e20250022.en

**Published:** 2026-05-22

**Authors:** Gabriela Breitenbach Santos, Iná da Silva dos Santos, Fernanda Pedrotti Moreira

**Affiliations:** 1 Universidade Católica de Pelotas Pelotas RS Brazil Universidade Católica de Pelotas, Pelotas, RS, Brazil; 2 Universidade Federal de Pelotas Pelotas RS Brazil Universidade Federal de Pelotas, Pelotas, RS, Brazil

**Keywords:** Hepatitis C, Liver Cirrhosis, Observational Study, Mass Screening, Therapeutics, Hepatitis C, Cirrosis Hepática, Estudio Observacional, Tamizaje Masivo, Terapéutica

## Abstract

**Objective:**

To describe the clinical-epidemiological profile of hepatitis C in individuals treated in Pelotas in 2023.

**Methods:**

This was a descriptive study, using data from medical records held at the Injectable Medication Administration and Follow-up Center. Characteristics of the individuals and the disease at the beginning of treatment were used, such as demographic variables, weight, origin of follow-up (public, private), viral load, genotyping, liver fibrosis staging, cirrhosis, human immunodeficiency virus coinfection, and treatment effectiveness. Analyses were stratified by sex, and differences were evaluated by Independent Samples t-test, Wilcoxon test or Fisher’s exact test.

**Results:**

102 individuals were followed up, i.e. 59 males and 43 females, with mean ages of 52.4 and 57.1 years (p-value 0.037). Human immunodeficiency virus coinfection was 5.9%. 35% of patients assessed underwent genotyping, with type 1 being the most frequent in males and type 3 in females. Adherence among public sector health professionals to the Ministry of Health liver fibrosis staging protocol was higher than among those working in the private sector. One male and six females presented an advanced degree of liver fibrosis (Aspartate Aminotransferase-to-Platelet Ratio Index ≥2) (p-value 0.049) at the start of treatment.

**Conclusion:**

Most patients treated for hepatitis C were referred by the public system, with a low degree of fibrosis, although one-third presented some degree of cirrhosis. Disease staging was assessed primarily among public system users. Females were the majority regarding being at an advanced stage of the natural history of the disease. Sustained virological response occurred predominantly among males.

## Resumen

**Objetivo:**

Describir el perfil clínico-epidemiológico de la hepatitis C en personas tratadas en Pelotas en 2023.

**Métodos:**

Estudio descriptivo, con datos de las historias clínicas del Centro de Aplicación y Monitoreo de Medicamentos Inyectables. Se analizaron las características de las personas y la enfermedad al inicio del tratamiento, como variables demográficas, peso, origen del seguimiento (público, privado), carga viral, genotipificación, estadificación de la fibrosis hepática, cirrosis, coinfección con el virus de la inmunodeficiencia humana y efectividad del tratamiento. Los análisis se estratificaron por sexo y las diferencias se evaluaron mediante la prueba t para muestras independientes, la prueba de Wilcoxon o la prueba exacta de Fisher.

**Resultados:**

Se realizó un seguimiento de 102 personas, 59 hombres y 43 mujeres, con edades medias de 52,4 y 57,1 años (valor p 0,037). La coinfección con el virus de la inmunodeficiencia humana fue del 5,9%. Al 35% de los pacientes evaluados se les realizó genotipificación, siendo el tipo 1 el más frecuente en hombres y el tipo 3 en mujeres. La adherencia al protocolo de estadificación de la fibrosis hepática establecido por el Ministerio de Salud entre los profesionales del sector público fue mayor que entre los del sector privado. Un hombre y seis mujeres presentaron un grado avanzado de fibrosis hepática (cociente aspartato aminotransferasa/plaquetas ≥2) (valor p 0,049) al inicio del tratamiento.

**Conclusión:**

La mayoría de los pacientes tratados por hepatitis C provenían del sistema público, con un bajo grado de fibrosis, aunque un tercio presentó algún grado de cirrosis. La estadificación de la enfermedad se evaluó principalmente entre los usuarios de este sistema. La mayoría de las mujeres se encontraban en una etapa avanzada de la historia natural de la enfermedad. La respuesta virológica sostenida se observó predominantemente en los hombres.

**Ethical aspects**

This research respected ethical principles, having obtained the following approval data:

Research ethics committee: Universidade Católica de Pelotas

Opinion number: 6,591,162

Approval date: 19/12/2023

Certificate of submission for ethical appraisal: 76438823.9.0000.5339

Informed consent form: Dismissed.

## Introduction

Viral hepatitis is responsible for 1.4 million deaths per year worldwide, due to acute infections, hepatocellular carcinoma and cirrhosis, and around half of these deaths are attributable to the hepatitis C virus (1). Hepatitis C virus infection causes acute and chronic hepatitis. Without treatment, within six months of infection, the virus may be eliminated spontaneously, but most affected individuals develop chronic disease, which can lead to progressive liver fibrosis and cirrhosis (2).

Once cirrhosis is established, the disease can remain indolent for many years or progress to hepatocellular carcinoma, liver decompensation, and death (3). From 2000 to 2022, 298,738 confirmed cases of hepatitis C were diagnosed in Brazil (4). The number of deaths caused by the hepatitis C virus dover the years in all regions of the country (4).

In 2016, the World Health Organization published the “Global Health Sector Strategy on Viral Hepatitis”, in which it recognized viral hepatitis (A, B, C, D and E) as an international public health problem (1). The main control strategies foreseen in that document are prevention of transmission through sexual contact and injectable drugs and expansion of treatment (1).

Regarding treatment of hepatitis C, there has been progress since 2013. This occurred after the introduction of direct-acting antiviral drugs, with which the sustained virological response increased from 50%, with the use of older drugs, to 95% efficacy against the hepatitis C virus (5-6). Sustained virological response is defined by the absence of detectable viral load after the infected person has started therapy (5).

In line with the World Health Organization’s strategy, a new protocol for managing hepatitis C was launched in Brazil in 2019 (2,7). This protocol includes the use of direct-acting antivirals, as well as the recommendation to treat all people with hepatitis C, regardless of the degree of fibrosis (7). Previously, this approach was reserved only for people with advanced fibrosis (7).

The national policy for controlling hepatitis C is based on broad screening of people at potential risk of infection with the virus (7). Testing is recommended for: people living with the human immunodeficiency virus; people with multiple partners or with sexually transmitted infections; people ≥40 years of age; dialysis patients; people who use alcohol and drugs; people deprived of liberty; people who received blood products before 1993; transplant recipients; people with percutaneous/parenteral exposure to biological materials that do not comply with health surveillance standards (individuals who have undergone tattoos and piercing implants or who use manicure services); health professionals; and people with diabetes, cardiovascular, liver or kidney diseases or with immunosuppression (7).

This study aimed to describe the clinical and epidemiological profile of hepatitis C in individuals treated in Pelotas in 2023.

## Methods

### Design

This is a descriptive study of the epidemiological and clinical data of patients treated for hepatitis C at the Pelotas Injectable Medication Administration and Follow-up Center.

### Setting

The data were extracted from the hepatitis C treatment request form, held on file at the Pelotas Injectable Medication Administration and Follow-up Center, a referral service for hepatitis C treatment for 12 municipalities in the region (Bagé, Amaral Ferrador, Arroio Grande, Arroio do Padre, Cerrito, Cristal, Jaguarão, Pedro Osório, Piratini, Pinheiro Machado, Santana da Boa Vista and Turuçu). 

In 2022, the Ministry of Health defined a new form to be completed by the attending physician and submitted to the Injectable Medication Administration and Follow-up Center for dispensing hepatitis C medication. In order to allow the health service time to adapt to the use of the new instrument, this study included individuals who started treatment in 2023. Data collection took place between January and September 2024.

### Participants

All individuals who started treatment for hepatitis C at the Pelotas Injectable Medication Administration and Follow-up Center between January 1 and December 31, 2023 were eligible for the study.

### Quantitative variables 

The individuals’ age was collected in as age at last birthday. The origin of follow-up was defined by the type of payment for medical care (public, private). The individuals’ weight was recorded in kilograms (kg), and the presence of chronic kidney disease (yes, no) was defined as creatinine clearance less than 30 milliliters/minute. Information was extracted on the occurrence of hepatitis B virus or human immunodeficiency virus coinfection. The hepatitis C viral load before treatment was classified into three groups: <500,000, 500,000-6 million, and >6 million international units (IU) per milliliter (ml). Although genotyping has not been mandatory for treatment requests since February 2022, the genotype found (type 1, 2, 3, 4, 5, and 6) was recorded for those who had undergone the test.

Liver fibrosis staging was performed using the Aspartate Aminotransferase-to-Platelet Ratio Index (APRI) (8), elastography (8-9), liver biopsy, or other methods (abdominal ultrasound or clinical findings of liver failure, such as ascites and esophageal varices). Interpretation of the fibrosis result determined that when APRI is <1, there is low probability of cirrhosis; when APRI is 1-1.49, it is not possible to determine the stage of liver fibrosis; and when APRI is ≥2, there is a high probability of cirrhosis (7). Degree of fibrosis was classified as F0 (no fibrosis), F1 (portal fibrosis without septa), F2 (few septa), F3 (numerous septa without cirrhosis), and F4 (cirrhosis) (10).

Severity of liver disease was defined by the presence or absence of cirrhosis. The Child-Pugh score (11) was used to identify individuals with decompensated cirrhosis. The score was based on total bilirubin levels, serum albumin, prothrombin time, ascites and symptoms of hepatic encephalopathy, ranging from five to 15 points. The Child-Pugh score is classified into three groups: Child-Pugh A (score of five to six, well-compensated disease), B (score of seven to nine, significant functional compromise), or C (score ≥10, decompensated disease). As per the treatment request form used in this study, Child-Pugh B and C were recorded as a single category.

The following information was extracted from the treatment-related information: whether previous treatment had been carried out for the current episode of hepatitis C (yes, no) and which medications had been prescribed (glecaprevir 100 mg/pibrentasvir 40 mg, ledispasvir 90 mg/sofosbuvir 400 mg, velpatasvir 100 mg/sofosbuvir 400 mg and/or ribavirin 250 mg) and duration of treatment. Treatment effectiveness was established according to sustained virological response (12), assessed through a genomic amplification test (polymerase chain reaction) on the individual’s plasma samples at the 12^th^ week after the end of drug therapy.

### Data sources 

The hepatitis C treatment request forms were reviewed at the Injectable Drug Application and Monitoring Center. Initially, the data were extracted, input and stored in a file specifically created for the study using Google Forms. They were then transferred to a Microsoft Excel spreadsheet (13), from where they were imported into Stata version 17.0 (14). Prior to analysis, the variables were edited in preparation for statistical analysis.

### Bias

As the data source was for clinical use, having been completed by the attending physicians of individuals referred to the Injectable Medication Administration and Follow-up Center, it was not possible to guarantee the reliability and completeness of all the information required on the individuals’ forms. On the other hand, there was no underreporting, since it was mandatory to present the form completed by the attending physician in order for the person to receive their medication.

### Study size 

The sample size calculation was performed using OpenEpi (12), taking the following parameters: population size equal to 150 (number of people having follow-up at the Injectable Drug Administration and Follow-up Center in 2022), 95% confidence interval (95% CI) and acceptable error of five percentage points, plus or minus. The largest sample size obtained was that required to identify people with hepatitis C who progressed to cirrhosis, estimated at 20% (3).

A further 10% was added to this number to account for possible incomplete records, resulting in a total of 104 people.

### Statistical methods

The analyses were descriptive. Initially, the distribution of the total sample was analyzed and, subsequently, stratified by sex. The difference between sexes regarding the means and proportion of the investigated characteristics was assessed using the Independent Samples t-test (when distribution was normal), the Wilcoxon test (for continuous values ​​without normal distribution), and Fisher’s exact test. Two-tailed p-values ​​less than 0.05 were considered statistically significant.

## Results

In all, 102 people were treated for hepatitis C in Pelotas in 2023. There were 3 deaths during the follow-up period: 1 after laboratory assessment for treatment control, 1 during treatment, and 1 after treatment but before the control test was performed.

Mean age was 54.4 years (standard deviation ±12.3 years), ranging from 23 to 89 years. The mean age of males (52.4 years; standard deviation ±10.8) was lower than that of females (57.1 years; standard deviation ±13.6; p-value 0.037). 87.0% of the participants had been referred by the public healthcare system, namely, 84.7% males and 90.7% females (p-value 0.549). The mean weight of males (76.8 kg) was higher than that of females (68.3 kg; p-value 0.016). Chronic kidney disease prevalence was 3.1%, and all cases (n=3) were found among males.

Human immunodeficiency virus coinfection prevalence was 5.9%, with no statistically significant difference between males (5.1%) and females (7.0%) (Table 1). No hepatitis C virus co-infection with hepatitis B virus was observed in the individuals assessed (data not shown in the table). Seventy-nine percent of participants had 500,000 to 6 million copies of the viral genome in their plasma at the start of treatment.

**Table 1:** Distribution of people with hepatitis C treated at the Injectable Medication Administration and Follow-up Center, according to clinical characteristics at the beginning of follow-up. Pelotas, 2023 (n=102)

Characteristic	Total	Male	Female	p-value
	n (%)	n (%)	n (%)	
**Coinfection **	6 (5.9)	3 (5.1)	3 (7.0)	0.695
**Viral load^a^** (UI/ml)				0.080
<500,000	13 (13.1)	4 (6.9)	9 (21.4)	
500,000-6 million	79 (79.0)	48 (82.8)	31 (73.8)	
>6 million	8 (8.0)	6 (10.3)	2 (4.8)	
Genotyping				0.145
Genotype 1	10 (9.8)	8 (13.6)	2 (4.6)	
Genotype 2	6 (5.9)	2 (3.4)	4 (9.3)	
Genotype 3	9 (8.8)	3 (5.1)	6 (13.9)	
Indeterminate	11 (10.8)	5 (8.5)	6 (13.9)	
Not performed	66 (64.7)	41 (69.5)	25 (58.1)	
**Aspartate Aminotransferase-to-Platelet Ratio Index**				0.105
<1.0	58 (56.9)	34 (57.6)	24 (55.8)	
1.0-1.9	13 (12.7)	9 (15.3)	4 (9.3)	
≥2.0	7 (6.9)	1 (1.7)	6 (14.0)	
Not performed	24 (23.5)	15 (25.4)	9 (20.9)	
**Elastography^b^**				0.400
F1	1 (1.0)	1 (1.7)	-	
F2	1 (1.0)	-	1 (2.3)	
F4	2 (2.0)	2 (3.4)	-	
Not performed	98 (96.0)	56 (95.0)	42 (97.7)	
**Other staging method^b^**				0.382
F0	2 (2.0)	2 (3.4)	-	
F4	5 (5.0)	4 (6.8)	1 (2.3)	
Not performed	95 (93.0)	53 (89.8)	42 (97.7)	
Severity^a^				0.382
No cirrhosis	65 (67.7)	35 (63.6)	30 (73.2)	
Child-Pugh A	23 (24.0)	16 (29.1)	7 (17.1)	
Child-Pugh B or C	8 (8.3)	4 (7.3)	4 (9.8)	

^a^Variables with less than 102 values due to missing information; ^b^F0 (no fibrosis), F1 (portal fibrosis without septa), F2 (few septa), F3 (numerous septa without cirrhosis) or F4 (cirrhosis) (10).

The proportion of males with a viral load greater than 6 million copies (10.3%) was double that observed among females (4.8%), but the difference was not statistically significant (p-value 0.080). Two-thirds of individuals (64.7%) did not have their samples submitted for viral genotyping, and the result was indeterminate for more than 10.0% of genotyped samples. Among individuals with valid results, the most frequent genotype among males was type 1 (13.6%) and type 3 (p-value 0.080) among females.

82.3% of the individuals underwent at least one examination to determine the stage of liver fibrosis. The Aspartate Aminotransferase-to-Platelet Ratio Index was the most frequently used examination (76.5%). The origin of medical follow-up was statistically associated with the performance of an examination for disease staging. Among public health sector service users, 85.4% underwent a liver fibrosis staging examination, while among private sector users this proportion was 61.5% (p-value 0.035).

Only 6.9% had a result ≥2 (p-value 0.105) among those assessed using the Aspartate Aminotransferase-to-Platelet Ratio Index. When only valid results (n=78) were counted and the variable was dichotomized (Aspartate Aminotransferase-to-Platelet Ratio Index ≥2, yes or no), the difference between males and females was statistically significant: 1 male (2.3%) and 6 females (17.7%) had an index ≥2 (p-value 0.039).

One third of the individuals (32.3%) had some degree of cirrhosis, and 23 of these 31 individuals were classified as Child A. There was no statistical difference between males and females regarding liver cirrhosis prevalence (p-value 0.323).

In the study population of 102 participants, one person had undergone prior treatment for hepatitis C. All received the sofosbuvir and velpatasvir treatment regimen, while for 2 people who had cirrhosis, ribavirin was included in their treatment. Prescribed treatment time was 12 weeks for 94.7% of males and 93.0% of females (Table 2). Sixty-six people (34 males and 32 females) returned with post-treatment test results; among these, 63 (95.5%) achieved a sustained virological response. Among males, sustained virological response prevalence was 97.1%, while among females it was 93.8% (p-value 0.519). Response to treatment was not dependent on the origin of medical follow-up referral (p-value 0.510), staging (p-value 0.224) or severity of liver disease (p-value 0.124) at the beginning of follow-up.

**Table 2:** Distribution of people with hepatitis C treated at the Injectable Medication Administration and Follow-up Center, according to length of treatment and sustained virological response. Pelotas, 2023 (n=102)

Characteristics	Total	Males	Females	p-value
	n (%)	n (%)	n (%)	
**Length of treatment **(weeks)				0.695
12	96 (94.0)	56 (94.9)	40 (93.0)	
24	6 (6.0)	3 (5.1)	3 (7.0)	
**Sustained virological response^a^**				0.519
Yes	63 (95.5)	33 (97.1)	30 (93.8)	
No	3 (4.5)	1 (2.9)	2 (6.2)	

^a^Variables with less than 102 values due to missing information.

## Discussion

In this study, individuals with hepatitis C treated in Pelotas in 2023 were, on average, in their sixth decade of life. There was a slight, but not statistically significant, predominance of males. The majority had been referred by the public health system. Disease staging was assessed more frequently among public sector users than among private sector users. When starting treatment, most individuals had a low degree of fibrosis. One-third of the participants had cirrhosis, most classified as Child A. The percentage of individuals with a high probability of cirrhosis was higher among females than among males. Only one person had received prior treatment for the current episode of hepatitis C, and all except one male and two females achieved a sustained virological response.

In a study in Brazil involving 532 people, conducted between 2015 and 2019, mean age was 56.8 years, with a slight predominance of males, these being results similar to those of this study (15). In an assessment of people with genotype 3 of the virus, carried out between 2011 and 2016 in Brazil, mean age was 59.3 years, with a similar frequency between males and females, and 56% of participants had cirrhosis, but more than 50% of participants had already been treated with other antiviral agents different from those currently in use (16). The findings of our study reflected the importance of initiating treatment in the early stages of the disease’s natural history, as well as the greater effectiveness of direct-acting antivirals (5-6). 

The similarity between mean ages in the three studies is noteworthy. In this study, there was a slight predominance of men treated for hepatitis C, as occurred in the United States between 2016 and 2017, where 55% of the participants in a given sample were male (19). In Turkey, between 2016 and 2021, 61% of the sample investigated was female, and in Taiwan in 2020, females accounted for 55% of the sample (17-18).

A large number of young people with tattoos has been observed in Brazil. A study conducted between 2018 and 2019 identified 13% of tattooed young people between 18-24 years old, 18% between 25-34 years old, and 14% between 35-44 years old (20). This is an eligibility criterion for hepatitis C screening, and in this study, the mean age of the participants was lower than that observed in Brazilian studies conducted before the new Ministry of Health protocol was launched in 2019. This finding possibly indicates the presence of hepatitis C under-screening in the southern region of Rio Grande do Sul state, where Pelotas is located. It is possible that only individuals at high risk of hepatitis C virus infection, and therefore for whom annual screening is recommendation, are being screened.

Taking a population of 424,644 inhabitants in 2022, over 20 years of age residing in Pelotas and the 12 municipalities covered by the Injectable Medication Administration and Follow-up Center (21) and with a national hepatitis C prevalence rate of 0.7% in 2017 (22), it can be estimated that there are 2,972 individuals living with the disease in the 12 municipalities served. Thus, the 102 people identified in 2023 suggest the occurrence of under-screening. The average number of annual medical consultations per adult recorded in Pelotas was 4.1 (95%CI 3.9; 4.3) (23). In 2007, numerous opportunities were missed for screening and identifying people who could benefit from early treatment, especially among females, as they consult most often (23).

Hepatitis C meets important criteria established by the World Health Organization for diseases appropriate for screening (1): it is serious, leads to liver failure and death; diagnostic testing is available and valid; treatment is effective and free; and prognosis is better if the disease is treated in earlier stages of its natural history. Studies specifically designed to assess healthcare professionals’ adherence to hepatitis C screening in the Southern region of Rio Grande do Sul state are highly necessary.

Regarding the severity of liver disease, in this study 32% of people presented cirrhosis. After the introduction of direct-acting antivirals in Brazil, it had been found that 54% of people had cirrhosis and, of these, 64% were classified as compensated cirrhotic individuals (Child A) (16). Most studies conducted outside Brazil found a variation of 30% to 60% of people with cirrhosis (24-25). The severity of cirrhotic people in studies conducted in other countries varied between 14% and 18% for compensated cirrhosis (Child A), and 9% and 12% for decompensated cirrhosis (Child B/C) (24,26-27).

In this study, 9% presented a high probability of cirrhosis. When only valid results were counted and the variable was dichotomized (Aspartate Aminotransferase-to-Platelet Ratio Index ≥2, yes or no), the difference between men and women was statistically significant, with a predominance of women. It had already been recorded that, with the exception of pregnancy, female sex was protective against the progression of hepatitis C. Women of reproductive age showed slower progression rates than men (in post-menopause, progression was similar between the sexes) (28).

In this study, 9% presented high probability of cirrhosis. The difference between males and females was only statistically significant when valid results were counted and the variable was dichotomized (Aspartate Aminotransferase-to-Platelet Ratio Index ≥2, yes or no), and in this cases females were predominant. It had been recorded previously that, with the exception of pregnancy, being of the female sex was protective against the progression of hepatitis C. Women of reproductive age showed slower progression rates than men (in the post-menopause, progression was similar between the sexes) (28).

The findings of this study suggested that the higher prevalence of fibrosis in the investigation is not related to the pathogenesis of hepatitis C. One hypothesis that may explain this result is the fact that sexually transmitted infections (eligibility criterion for screening) were recorded as more asymptomatic in females (29), which would lead to greater under-screening among this population than among males.

Adherence among public sector health professionals to the Ministry of Health liver fibrosis staging protocol was higher than among those working in the private sector. No other findings were found regarding professional adherence according to the type of consultation funding. An assessment of health professional adherence to lipid profile screening in Pelotas demonstrated that, in relation to the private/health insurance sector, the public sector had a greater focus (74.7% versus 62.3%; p-value<0.001) and a higher screening ratio (division between the percentage of screening requested for people who met the screening criteria and the percentage requested for those who did not meet the criteria), with 1.97 in the public sector and 1.46 in the private/health insurance sector (30).

This study had both positive and limited aspects. Among the positive aspects, the sample studied corresponded to all individuals treated at the Injectable Medication Administration and Follow-up Center in 2023, originating from Pelotas and other municipalities in the region. There was also no underreporting of individuals, since it was mandatory to present the form completed by the attending physician in order for the person to receive their medication.

Among the limitations, the small sample size did not allow for the identification of statistical differences between sexes or regarding the age of the participants. It was not possible to assess the participants’ adherence to the therapeutic regimen. Sustained virological response was only verifiable for 68 people; in addition to the two deaths before the examination was performed, 30 people did not return to obtain the request for the control examination, and two withdrew the examination request and did not return with the result. The lack of information on genotyping and the indeterminate result among those who underwent this examination limited the study’s ability to assess the prevalence of different viral genotypes.

In conclusion, the mean age of the participants was 54.4 years, most came from the public healthcare system, presented low-grade fibrosis, and one-third had some degree of cirrhosis. Disease staging was more pronounced among public sector users. There were more females than males in the more advanced stages of the natural history of the disease. A sustained virological response occurred in most individuals.

The study raised the hypothesis of possible hepatitis C under-screening, mainly among females. In order to meet the Ministry of Health’s goals regarding identification and management of people with the hepatitis C virus, it is necessary for health professionals to adhere to disease screening, identifying infected individuals who are still asymptomatic and potential transmitters. This allows for more effective control of the agent’s transmission and brings forward the start of treatment for those infected.

## Data Availability

The database and analysis codes used in this research are available at: https://drive.google.com/drive/folders/1XJMCIliVhAMVorBFFXKAZ8Dmt_tD6shA?usp=sharing.
