# Supplementary material for: Profile of people treated for hepatitis C: a descriptive study, Pelotas, Brazil, 2023
Source: Epidemiol Serv Saude. 2026 May 22;35:e20250022. doi: 10.1590/S2237-96222026v35e20250022.en (PMC13686298; doi:10.1590/S2237-96222026v35e20250022.en)
Supplement: Translation

# Translation: Perfil das pessoas tratadas para hepatite C: estudo descritivo, Pelotas, 2023

**DOI:** 10.1590/S2237-96222026v35e20250022.pt

**Authors:** Gabriela Breitenbach Santos, Iná da Silva dos Santos, Fernanda Pedrotti Moreira

**Affiliations:** 1 Universidade Católica de Pelotas Pelotas RS Brasil Universidade Católica de Pelotas, Pelotas, RS, Brasil; 2 Universidade Federal de Pelotas Pelotas RS Brasil Universidade Federal de Pelotas, Pelotas, RS, Brasil

**Keywords:** Covid-19, Hospitalização, Mortalidade, Estudos de Coorte, Brasil

## Abstract

**Objetivo:**

Descrever o perfil clínico-epidemiológico da hepatite C em indivíduos tratados em Pelotas em 2023.

**Métodos:**

Estudo descritivo, com dados dos prontuários do Centro de Aplicação e Monitoramento de Medicamentos Injetáveis. Utilizaram-se características das pessoas e da doença no início do tratamento, como variáveis demográficas, peso, origem do acompanhamento (público, privado), carga viral, genotipagem, estadiamento da fibrose hepática, cirrose, coinfecção com vírus da imunodeficiência humana e efetividade do tratamento. As análises foram estratificadas por sexo, e as diferenças avaliadas por teste T para amostras independentes, Wilcoxon ou exato de Fisher.

**Resultados:**

Acompanharam-se 102 pessoas, sendo 59 homens e 43 mulheres, com médias de idade de 52,4 e de 57,1 anos (p-valor 0,037). A coinfecção com vírus da imunodeficiência humana foi 5,9%. 35% dos pacientes avaliados fizeram genotipagem, o tipo mais frequente em homens, e o tipo 3, em mulheres. A adesão dos profissionais do setor público ao protocolo de estadiamento da fibrose hepática estabelecido pelo Ministério de Saúde foi maior do que entre os do privado. Um homem e seis mulheres apresentavam grau avançado de fibrose hepática (índice de relação aspartato aminotransferase sobre plaquetas ≥2) (p-valor 0,049) ao iniciar o tratamento.

**Conclusão:**

A maioria dos pacientes tratados para hepatite C vinha do sistema público, com baixo grau de fibrose, embora um terço apresentasse algum grau de cirrose. O estadiamento da doença foi avaliado sobretudo entre usuários desse sistema. Houve maioria de mulheres em fase adiantada da história natural da doença. A resposta virológica sustentada ocorreu majoritariamente entre os indivíduos.

**Aspectos éticos**

Esta pesquisa respeitou os princípios éticos, obtendo os seguintes dados de aprovação: :

Comitê de ética em pesquisa: Universidade Católica de Pelotas

Número do parecer: 6.591.162

Data de aprovação: 19/12/2023

Certificado de apresentação de apreciação ética: 76438823.9.0000.5339

Registro do consentimento livre e esclarecido: Dispensado.

## Introdução

As hepatites virais são responsáveis por 1,4 milhão de mortes por ano no mundo, devido a infecções agudas, hepatocarcinoma e cirrose, e cerca de metade dessas mortes são atribuíveis ao vírus da hepatite C (1). A infecção pelo vírus da hepatite C causa hepatite aguda e crônica. Na ausência de tratamento, no período de seis meses após a infecção, a eliminação do vírus pode ocorrer espontaneamente, mas a maioria dos afetados desenvolve a doença crônica, que pode levar à fibrose progressiva do fígado e à cirrose (2). 

Uma vez estabelecida a cirrose, a doença pode permanecer indolente durante muitos anos ou progredir para carcinoma hepatocelular, descompensação hepática e morte (3). De 2000 a 2022, foram diagnosticados no Brasil 298.738 casos confirmados de hepatite C (4). O número de óbitos causados pelo vírus da hepatite C aumenta ao longo dos anos em todas as regiões do país (4).

Em 2016, a Organização Mundial de Saúde publicou a “Global Health Sector Strategy on Viral Hepatitis”, na qual reconheceu as hepatites virais (A, B, C, D e E) como problema de saúde pública internacional (1). As principais estratégias de controle previstas nesse documento são a prevenção da transmissão por via sexual e drogas injetáveis e a ampliação do tratamento (1). 

Em relação ao tratamento da hepatite C, houve avanços desde 2013. Isso ocorreu após a introdução dos medicamentos antivirais de ação direta, com os quais a resposta virológica sustentada subiu de 50%, com o uso dos medicamentos antigos, para 95% de eficácia contra o vírus da hepatite C (5-6). A resposta virológica sustentada é definida pela ausência de carga viral detectável após iniciada a terapia do infectado (5). 

Alinhado à estratégia da Organização Mundial de Saúde, em 2019, foi lançado no Brasil um novo protocolo para manejo da hepatite C (2,7). Esse protocolo inclui o uso dos antivirais de ação direta, além da recomendação do tratamento de todas as pessoas com hepatite C, independentemente do grau de fibrose (7). Anteriormente, essa conduta era reservada apenas para pessoas com fibrose avançada (7). 

A política nacional de controle da hepatite C é baseada na triagem ampla de pessoas em risco potencial de infecção pelo vírus (7). É recomendada a testagem em: pessoas que vivem com o vírus da imunodeficiência humana; pessoas com múltiplos parceiros ou com infecções sexualmente transmissíveis; pessoas com ≥40 anos de idade; dialíticos; pessoas que fazem uso de álcool e drogas; pessoas privadas de liberdade; pessoas que receberam hemoderivados antes de 1993; transplantados; pessoas com exposição percutânea/parenteral a materiais biológicos que não obedeçam às normas da vigilância sanitária (indivíduos que se submeteram a tatuagens e implantes de piercings ou que utilizam serviços de manicure); profissionais de saúde; e pessoas com diabetes, doenças cardiovasculares, hepáticas ou renais ou com imunodepressão (7). 

Este estudo teve como objetivo descrever o perfil clínico-epidemiológico da hepatite C em indivíduos tratados em Pelotas, em 2023. 

## Métodos

### Delineamento

Trata-se de estudo descritivo dos dados epidemiológicos e clínicos de pacientes tratados para hepatite C no Centro de Aplicação e Monitoramento de Medicamentos Injetáveis de Pelotas.

### Contexto

Os dados foram extraídos do formulário de solicitação de tratamento para hepatite C, arquivados no Centro de Aplicação e Monitoramento de Medicamentos Injetáveis de Pelotas, referência para o tratamento de hepatite C de 12 municípios da região (Bagé, Amaral Ferrador, Arroio Grande, Arroio do Padre, Cerrito, Cristal, Jaguarão, Pedro Osório, Piratini, Pinheiro Machado, Santana da Boa Vista e Turuçu). 

Em 2022, o Ministério da Saúde definiu novo formulário a ser preenchido pelo médico assistente e apresentado ao Centro de Aplicação e Monitoramento de Medicamentos Injetáveis para dispensação de medicamentos para hepatite C. A fim de dar tempo ao serviço de saúde de se adequar ao uso do novo instrumento, foram incluídas no estudo as pessoas que iniciaram tratamento em 2023. A coleta de dados ocorreu entre janeiro e setembro de 2024.

### Participantes

Todas as pessoas que iniciaram tratamento para hepatite C no Centro de Aplicação e Monitoramento de Medicamentos Injetáveis de Pelotas entre 1º de janeiro e 31 de dezembro de 2023 foram elegíveis para o estudo.

### Variáveis quantitativas

A idade das pessoas foi coletada em anos completos. A origem do acompanhamento referiu-se à forma de pagamento do cuidado médico (público, privado). O peso das pessoas foi registrado em quilogramas (kg), e a presença de doença renal crônica (sim, não) foi definida como depuração de creatinina menor de 30 mililitros/minuto. Foi extraída informação sobre a ocorrência de coinfecção com vírus da hepatite B ou vírus da imunodeficiência humana. A carga viral do vírus da hepatite C antes do tratamento foi classificada em três grupos: <500 mil, 500 mil-6 milhões e >6 milhões de unidades internacionais (UI) por mililitro (ml). Embora desde fevereiro de 2022 a genotipagem não seja obrigatória para solicitação do tratamento, o genótipo encontrado (tipo 1, 2, 3, 4, 5 e 6) foi registrado para os que haviam feito o exame. 

O estadiamento da fibrose hepática foi realizado por meio do índice de relação aspartato aminotransferase sobre plaquetas (8), elastografia (8-9), biópsia hepática ou outro método (ultrassom abdominal ou clínica de insuficiência hepática, como ascite e varizes esofágicas). A interpretação do resultado quanto à fibrose determinou que, quando o índice de relação aspartato aminotransferase sobre plaquetas for <1, há baixa probabilidade de cirrose; quando o índice de relação aspartato aminotransferase sobre plaquetas for 1-1,49, não é possível determinar o estágio de fibrose hepática; e quando o índice de relação aspartato aminotransferase sobre plaquetas for ≥2, existe alta probabilidade de cirrose (7). O grau de fibrose foi classificado em F0 (sem fibrose), F1 (fibrose portal sem septos), F2 (poucos septos), F3 (numerosos septos sem cirrose) e F4 (cirrose) (10). 

A gravidade da hepatopatia foi definida pela presença ou pela ausência de cirrose. O escore de Child-Pugh (11) foi empregado para identificar as pessoas com cirrose descompensada. O escore baseou-se nos níveis de bilirrubinas totais, albumina sérica, tempo de protrombina, ascite e sintomas de encefalopatia hepática, com variação de cinco a 15 pontos. O escore Child-Pugh é classificado em três grupos: Child-Pugh A (escore de cinco a seis, doença bem compensada), B (escore de sete a nove, comprometimento funcional significativo) ou C (escore ≥10, doença descompensada). Conforme o formulário da requisição do tratamento utilizado nesse estudo, Child-Pugh B e C eram registrados como uma única categoria.

Das informações relacionadas ao tratamento, foram extraídas: se havia sido realizado tratamento prévio para o atual episódio de hepatite C (sim, não) e quais foram os medicamentos prescritos (glecaprevir 100 mg/pibrentasvir 40 mg, ledispasvir 90 mg/sofosbuvir 400 mg, velpatasvir 100 mg/sofosbuvir 400 mg e/ou ribavirina 250 mg) e a duração do tratamento. A efetividade do tratamento foi estabelecida de acordo com a resposta virológica sustentada (12), avaliada através de um teste de amplificação genômica (reação em cadeia pela polimerase) em amostras de plasma da pessoa na 12ª semana após o término da terapia medicamentosa.

### Fontes de dados 

Os formulários de requisição de tratamento para hepatite C foram revisados nas dependências do Centro de Aplicação e Monitoramento de Medicamentos Injetáveis. Os dados foram extraídos, digitados e armazenados em arquivo especialmente construído para o estudo no Google Forms; depois, transferidos para planilha do Microsoft Excel (13), de onde foram importados para o programa Stata versão 17.0 (14). Antes de serem analisadas, as variáveis foram editadas para se adequarem à análise estatística.

### Viés

Como a fonte de dados foi de uso clínico, tendo sido preenchida pelos médicos assistentes das pessoas encaminhadas ao Centro de Aplicação e Monitoramento de Medicamentos Injetáveis, não foi possível garantir a fidedignidade e a completude de todas as informações requeridas nos formulários das pessoas. Por outro lado, não houve sub-registro, uma vez que era obrigatória a apresentação do formulário preenchido pelo médico assistente para que a pessoa pudesse receber o medicamento.

### Tamanho do estudo

O cálculo do tamanho amostral foi realizado no programa OpenEpi (12), com a fixação dos seguintes parâmetros: tamanho da população igual a 150 (número de pessoas acompanhadas no Centro de Aplicação e Monitoramento de Medicamentos Injetáveis em 2022), intervalo de confiança de 95% (IC95%) e erro aceitável de cinco pontos percentuais, para mais ou para menos. O maior tamanho amostral obtido foi o necessário para identificar pessoas com hepatite C que evoluíram para cirrose, estimado em 20% (3). Seria necessário incluir, então, 94 pessoas. A esse valor foi adicionado 10%, para possíveis registros incompletos, chegando-se a 104 pessoas. 

### Métodos estatísticos

As análises foram descritivas. Inicialmente, foi analisada a distribuição da amostra total e, posteriormente, estratificada por sexo. A diferença entre os sexos quanto às médias e à proporção das características investigadas foi avaliada pelos testes T para amostras independentes (quando a distribuição era normal), de Wilcoxon (para valores contínuos sem distribuição normal) e exato de Fisher. Valores p bicaudais menores que 0,05 foram considerados estatisticamente significativos.

## Resultados

Ao todo, 102 pessoas foram tratadas para hepatite C em Pelotas em 2023. Houve 3 óbitos ao longo do acompanhamento: 1 após a avaliação laboratorial para controle de tratamento, 1 durante o tratamento e 1 após o tratamento, mas antes da realização do exame de controle. 

A média de idade foi 54,4 anos (desvio-padrão±12,3 anos), com variação entre 23 e 89 anos. A média de idade dos homens (52,4 anos; desvio-padrão±10,^8^) foi menor que a das mulheres (57,1 anos; desvio-padrão±13,6; p-valor 0,037). Eram provenientes do sistema público 87,0% das pessoas, 84,7% entre os homens e 90,7% entre as mulheres (p-valor 0,549). A média de peso dos homens (76,8 kg) foi maior que a das mulheres (68,3 kg; p-valor 0,016). A prevalência de doença renal crônica foi de 3,1%, e todos os casos (n=3) foram encontrados entre os homens. 

A prevalência de coinfecção com vírus da imunodeficiência humana foi de 5,9%, sem diferença estatística entre homens (5,1%) e mulheres (7,0%) (Tabela 1). Não foi observada coinfecção do vírus da hepatite C com vírus da hepatite B nas pessoas avaliadas (dados não mostrados na tabela). Setenta e nove por cento dos participantes apresentavam 500 mil a 6 milhões de cópias do genoma viral no plasma ao iniciarem o tratamento. 

**Tabela 1:** Distribuição das pessoas com hepatite C tratadas no Centro de Aplicação e Monitoramento de Medicamentos Injetáveis, de acordo com características clínicas no início do acompanhamento. Pelotas, 2023 (n=102)

Característica	Todos	Homens	Mulheres	p-valor
	n (%)	n (%)	n (%)	
**Coinfecção **	6 (5,9)	3 (5,1)	3 (7,0)	0,695
**Carga virala **(UI/ml)				0,080
<500 mil	13 (13,1)	4 (6,9)	9 (21,4)	
500 mil-6 milhões	79 (79,0)	48 (82,8)	31 (73,8)	
>6 milhões	8 (8,0)	6 (10,3)	2 (4,8)	
Genotipagem				0,145
Genótipo 1	10 (9,8)	8 (13,6)	2 (4,6)	
Genótipo 2	6 (5,9)	2 (3,4)	4 (9,3)	
Genótipo 3	9 (8,8)	3 (5,1)	6 (13,9)	
Indeterminado	11 (10,8)	5 (8,5)	6 (13,9)	
Não fez	66 (64,7)	41 (69,5)	25 (58,1)	
**Índice de relação aspartato aminotransferase sobre plaquetas**				0,105
<1,0	58 (56,9)	34 (57,6)	24 (55,8)	
1,0-1,9	13 (12,7)	9 (15,3)	4 (9,3)	
≥2,0	7 (6,9)	1 (1,7)	6 (14,0)	
Não fez	24 (23,5)	15 (25,4)	9 (20,9)	
**Elastografia^b^**				0,400
F1	1 (1,0)	1 (1,7)	-	
F2	1 (1,0)	-	1 (2,3)	
F4	2 (2,0)	2 (3,4)	-	
Não fez	98 (96,0)	56 (95,0)	42 (97,7)	
**Outro método de estadiamento^b^**				0,382
F0	2 (2,0)	2 (3,4)	-	
F4	5 (5,0)	4 (6,8)	1 (2,3)	
Não fez	95 (93,0)	53 (89,8)	42 (97,7)	
Gravidade^a^				0,382
Sem cirrose	65 (67,7)	35 (63,6)	30 (73,2)	
Child-Pugh A	23 (24,0)	16 (29,1)	7 (17,1)	
Child-Pugh B ou C	8 (8,3)	4 (7,3)	4 (9,8)	

^a^Variáveis com menos de 102 valores devido à perda de informação; ^b^F0 (sem fibrose), F1 (fibrose portal sem septos), F2 (poucos septos), F3 (numerosos septos sem cirrose) ou F4 (cirrose) (10).

A proporção de homens com carga viral superior a 6 milhões de cópias (10,3%) foi o dobro da observada entre as mulheres (4,8%), mas a diferença não foi estatisticamente significativa (p-valor 0,080). Dois terços das pessoas (64,7%) não tiveram suas amostras submetidas à genotipagem viral e, em mais de 10,0% daquelas amostras genotipadas, o resultado foi indeterminado. Entre as pessoas com resultados válidos, o genótipo mais frequente entre os homens foi o tipo 1 (13,6%) e, entre as mulheres, o tipo 3 (p-valor 0,080). 

Foram submetidas a pelo menos um exame para definir o estadiamento da fibrose hepática 82,3% das pessoas. O índice de relação aspartato aminotransferase sobre plaquetas foi o mais empregado (76,5%). A origem do acompanhamento médico associou-se estatisticamente com a realização de exame para estadiamento da doença. Entre os usuários originários do setor público, 85,4% realizaram exame de estadiamento da fibrose hepática. Entre os provenientes do setor privado, essa proporção foi de 61,5% (p-valor 0,035).

Apenas 6,9% apresentavam resultado ≥2 (p-valor 0,105) entre os que foram avaliados pelo índice de relação aspartato aminotransferase sobre plaquetas. Quando apenas os resultados válidos (n=78) foram contabilizados e a variável foi dicotomizada (índice de relação aspartato aminotransferase sobre plaquetas ≥2, sim ou não), a diferença entre homens e mulheres foi estatisticamente significativa: 1 homem (2,3%) e 6 mulheres (17,7%) tiveram índice ≥2 (p-valor 0,039). 

Um terço das pessoas (32,3%) apresentava algum grau de cirrose, e 23 das 31 pessoas foram classificados como Child A. Não houve diferença estatística entre homens e mulheres quanto à prevalência de cirrose hepática (p-valor 0,323).

Na população estudada de 102 participantes, uma pessoa havia realizado tratamento prévio para hepatite C. Todos receberam o esquema de tratamento com sofosbuvir e velpatasvir, e, para 2 pessoas que apresentavam cirrose, a ribavirina foi incluída no tratamento. Para 94,7% dos homens e 93,0% das mulheres, o tempo de tratamento prescrito foi de 12 semanas (Tabela 2). Sessenta e seis pessoas (34 homens e 32 mulheres) retornaram com resultado de exames pós-tratamento; entre esses, 63 (95,5%) atingiram resposta virológica sustentada. Entre os homens, a prevalência de resposta virológica sustentada foi de 97,1% e, entre as mulheres, de 93,8% (p-valor 0,519). A resposta ao tratamento foi independente da origem do acompanhamento médico (p-valor 0,510), do estadiamento (p-valor 0,224) ou da gravidade da doença hepática (p-valor 0,124) no início do acompanhamento. 

**Tabela 2:** Distribuição das pessoas com hepatite C tratadas no Centro de Aplicação e Monitoramento de Medicamentos Injetáveis, de acordo com o tempo de tratamento e resposta viral sustentada. Pelotas, 2023 (n=102)

Características	Todos	Homens	Mulheres	p-valor
	n (%)	n (%)	n (%)	
**Tempo de tratamento **(semanas)				0,695
12	96 (94,0)	56 (94,9)	40 (93,0)	
24	6 (6,0)	3 (5,1)	3 (7,0)	
**Resposta viral sustentada^a^**				0,519
Sim	63 (95,5)	33 (97,1)	30 (93,8)	
Não	3 (4,5)	1 (2,9)	2 (6,2)	

^a^Variáveis com menos de 102 valores devido à perda de informação.

## Discussão

Neste estudo, as pessoas com hepatite C tratadas em Pelotas em 2023 estavam, em média, na sexta década da vida. Havia leve predomínio, não estatisticamente significativo, do sexo masculino. A maioria era proveniente do sistema público de saúde. O estadiamento da doença foi avaliado mais frequentemente entre os usuários do setor público do que entre os do setor privado. Ao se apresentarem para o tratamento, a maioria das pessoas tinha baixo grau de fibrose. Um terço dos participantes apresentava cirrose, a maioria classificada como Child A. O percentual de pessoas com alta probabilidade de cirrose foi maior entre as mulheres que entre os homens. Apenas uma pessoa havia realizado tratamento prévio para o atual episódio de hepatite C e todos, exceto um homem e duas mulheres, alcançaram resposta virológica sustentada.

Em investigação no Brasil com 532 pessoas, realizada entre 2015 e 2019, a média de idade foi 56,8 anos, com discreto predomínio de homens, resultados semelhantes aos deste estudo (15). Em avaliação de pessoas com genótipo 3 do vírus, realizada entre 2011 e 2016 no país, a média de idade foi 59,3 anos, com frequência semelhante entre homens e mulheres, e 56% das pessoas apresentavam cirrose, porém mais de 50% dos participantes já haviam sido tratados com outros agentes antivirais diferentes dos atualmente em uso (16). Os achados deste estudo refletiram a importância de iniciar o tratamento na fase inicial da história natural da doença, bem como a maior efetividade dos antivirais de ação direta (5-6). Chama a atenção a semelhança entre as médias de idade nos três estudos. 

Neste estudo, houve discreto predomínio de homens tratados com hepatite C, conforme o ocorrido nos Estados Unidos entre 2016 e 2017, em que 55% dos participantes de determinada amostra eram do sexo masculino (19). Na Turquia, entre 2016 e2021, 61% de amostra investigada era de mulheres e, em Taiwan em 2020, o sexo feminino representou 55% da amostra (17-18).

Tem-se observado grande número de jovens com tatuagem no Brasil. Foram identificados, em estudo realizado entre 2018 e 2019, 13% de jovens tatuados entre 18-24 anos, 18% entre 25-34 anos e 14% entre 35-44 anos (20). Esse é um critério de elegibilidade para triagem de hepatite C e, neste estudo, a média de idade das pessoas foi menor do que a observada em estudos brasileiros realizados antes que o novo protocolo do Ministério da Saúde fosse lançado em 2019. Esse achado possivelmente indica a presença de sub-rastreio de hepatite C na região Sul do Rio Grande do Sul. É possível que apenas indivíduos com alto risco de infecção pelo vírus da hepatite C e, portanto, com recomendação de rastreio anual, sejam rastreados. 

Com uma população de 424.644 habitantes, em 2022, maiores de 20 anos de idade residentes em Pelotas e nos 12 municípios cobertos pelo Centro de Aplicação e Monitoramento de Medicamentos Injetáveis (21) e com uma prevalência nacional de hepatite C de 0,7% em 2017 (22), é de esperar que haja 2.972 indivíduos vivendo com a doença nos 12 municípios atendidos. Assim, as 102 pessoas identificadas em 2023 sugerem ocorrência de sub-rastreio. Foi registrado que o número médio de consultas médicas anuais por adultos em Pelotas era de 4,1 (IC95% 3,9; 4,3) (23). Em 2007, inúmeras oportunidades foram perdidas de rastrear e identificar pessoas que poderiam se beneficiar do tratamento precoce, principalmente entre as mulheres, as pessoas que mais consultam (23). 

A hepatite C atende a importantes critérios estabelecidos pela Organização Mundial da Saúde de doenças apropriadas para rastreio (1): é grave, leva à insuficiência hepática e à morte; o exame diagnóstico está disponível e é válido; o tratamento é eficaz e gratuito; e o prognóstico é melhor se a doença for tratada em fases mais precoces da história natural. Estudos especificamente planejados para avaliar a adesão dos profissionais ao rastreio da hepatite C na região Sul do Rio Grande do Sul são altamente necessários.

Quanto à gravidade da hepatopatia, neste estudo 32% pessoas apresentaram cirrose. Após a introdução dos antivirais de ação direta no Brasil, havia sido constatado que 54% das pessoas apresentavam cirrose e, dessas, 64% foram classificadas como cirróticos compensados (Child A) (16). A maioria dos estudos conduzidos no exterior encontrou variação de 30% a 60% de pessoas com cirrose (24-25). A gravidade das pessoas cirróticos nos estudos conduzidos em outros países variou entre 14% e 18%, para cirrose compensada (Child A), e 9% e 12%, para cirrose descompensada (Child B/C) (24,26-27). 

Neste estudo, 9% apresentaram alta probabilidade de cirrose. Quando apenas os resultados válidos foram contabilizados e a variável foi dicotomizada (índice de relação aspartato aminotransferase sobre plaquetas ≥2, sim ou não), a diferença entre homens e mulheres foi estatisticamente significativa, com predomínio das mulheres. Já havia sido registrado que, com a exceção da gestação, o sexo feminino foi protetor em relação à progressão da hepatite C. As mulheres em idade reprodutiva apresentaram taxas de progressão mais lentas do que a dos homens (na pós-menopausa, a progressão foi similar entre os sexos) (28). 

Os achados destes estudo sugeriram que a maior prevalência da fibrose na investigação não está relacionada com a patogênese da hepatite C. Uma das hipóteses para explicar esse resultado é o fato de que as doenças de transmissão sexual (critério de elegibilidade para rastreio) foram registradas como mais assintomáticas em mulheres (29), o que levaria ao maior sub-rastreio entre essa população do que entre os homens.

A adesão dos profissionais do setor público ao protocolo de estadiamento da fibrose hepática estabelecido pelo Ministério de Saúde foi maior do que entre os do setor privado. Não foram localizados outros achados sobre a adesão do profissional conforme o tipo de financiamento da consulta. Em avaliação acerca da adesão dos profissionais ao rastreio do perfil lipídico em Pelotas, foi demonstrado que, em relação ao privado/convênio, o setor público teve maior foco (74,7% versus 62,3%; p-valor<0,001) e maior razão de rastreio (divisão entre o percentual do rastreio solicitado para pessoas que atendiam ao critério de rastreio e o percentual solicitado para quem não atendia), com 1,97 no setor público e 1,46 no privado/convênio (30).

Este estudo teve aspectos positivos e limitações. Entre os positivos, a amostra estudada correspondeu à totalidade das pessoas atendidas no Centro de Aplicação e Monitoramento de Medicamentos Injetáveis em 2023, oriundos de Pelotas e de outros municípios da região. Também não houve sub-registro de pessoas, uma vez que era obrigatória a apresentação do formulário preenchido pelo médico assistente para que a pessoa pudesse receber o medicamento. 

Entre as limitações, o pequeno tamanho da amostra não permitiu identificar diferenças estatísticas entre os sexos ou quanto à idade das pessoas. Não foi possível avaliar a adesão das pessoas ao regime terapêutico. A resposta virológica sustentada só foi verificável para 68 pessoas; para além dos dois óbitos antes da realização do exame, 30 pessoas não retornaram para obter a solicitação do exame de controle, e duas retiraram a solicitação do exame e não retornaram com o resultado. A falta de informação sobre genotipagem e o resultado indeterminado entre os que fizeram esse exame limitaram a capacidade do estudo em avaliar a prevalência dos diferentes genótipos do vírus.

Em conclusão, as pessoas tinham em média 54,4 anos, a maioria proveniente do sistema público, com baixo grau de fibrose e um terço apresentou algum grau de cirrose. O estadiamento da doença foi mais avaliado entre usuários do setor público. Havia mais mulheres do que homens em fase mais adiantada da história natural da doença. A resposta virológica sustentada ocorreu na maioria das pessoas. 

O estudo levantou a hipótese de possível sub-rastreio de hepatite C, principalmente entre as mulheres. Para atender às metas do Ministério da Saúde no que tange à identificação e ao manejo de pessoas com o vírus da hepatite C, é necessária adesão dos profissionais ao rastreio da doença, com a identificação dos indivíduos infectados ainda assintomáticos, os quais são potencialmente transmissores. Isso propicia maior controle efetivo da transmissão do agente e antecipa o início do tratamento dos infectados.

## Data Availability

O banco de dados e os códigos de análise utilizados na pesquisa estão disponíveis em: https://drive.google.com/drive/folders/1XJMCIliVhAMVorBFFXKAZ8Dmt_tD6shA?usp=sharing.
